# Resting Heart Rate as a Predictor of Cancer Mortality: A Systematic Review and Meta-Analysis

**DOI:** 10.3390/jcm10071354

**Published:** 2021-03-25

**Authors:** Diana P. Pozuelo-Carrascosa, Iván Cavero-Redondo, I.M. Lee, Celia Álvarez-Bueno, Sara Reina-Gutierrez, Vicente Martínez-Vizcaíno

**Affiliations:** 1 Health and Social Care Research Center, Universidad de Castilla-La Mancha, 16071 Cuenca, Spain; dianap.pozuelo@uclm.es (D.P.P.-C.); celia.alvarezbueno@uclm.es (C.Á.-B.); sara.reina@uclm.es (S.R.-G.); vicente.martinez@uclm.es (V.M.-V.); 2 Faculty of Physiotherapy and Nursing, Universidad de Castilla-La Mancha, 45004 Toledo, Spain; 3 Rehabilitation in Health Research Center (CIRES), Universidad de las Americas, Echaurren Street 140, 2nd Floor, 72819 Santiago, Chile; 4 Department of Epidemiology, Harvard T.H. Chan School of Public Health, Boston, MA 02115, USA; ilee@rics.bwh.harvard.edu; 5 Division of Preventive Medicine, Brigham and Women’s Hospital, Harvard Medical School, Boston, MA 02115, USA; 6 Universidad Politécnica y Artística del Paraguay, Asunción 001518, Paraguay; 7 Faculty of Health Sciences, Universidad Autónoma de Chile, 1670 Talca, Chile

**Keywords:** cancer mortality, resting heart rate, meta-analysis, risk of cancer

## Abstract

This work was aimed to synthetize the evidence available about the relationship between resting heart rate (RHR) and the risk of cancer mortality. A computerized search in the Medline, EMBASE, Web of Science, and Cochrane Library databases from their inception to 24 September 2020 was performed. We performed three meta-analyses: (1) cancer mortality comparing the “less than 60 bpm” and “more than 60 bpm” categories; (2) cancer mortality comparing “less than 60 bpm”, “60 to 80 bpm”, and “more than 80 bpm” categories; and (3) analysis for 10–12 and 20 bpm increase in RHR and risk of cancer mortality. Twenty-two studies were included in the qualitative review, and twelve of them met the inclusion criteria for the meta-analysis. Our results showed a positive association between RHR and the risk of cancer mortality. This association was shown in a meta-analysis comparing studies reporting mean RHR values below and above 60 bpm, when comparing three RHR categories using less than 60 bpm as the reference category and, finally, in dose response analyses estimating the effect of an increase of 10–12 bpm in RHR, both in men and in women. In conclusion, a low RHR is a potential marker of low risk of cancer mortality.

## 1. Introduction

Resting heart rate (RHR) is a fundamental clinical marker and an indicator of health or disease; in fact, high RHR is usually related to disease, and a low RHR is associated with better health [1]. Assessing heart rate is a classical procedure of physical evaluation, and the measurement of the RHR is a simple and inexpensive procedure that can be easily performed in routine clinical practice.

The etiology of cancer involves, in addition to genetic factors, some environmental/lifestyle risk factors, such as physical activity, that are associated with the incidence of this disease [2,3]. Among other effects derived from the practice of physical activity is a decrease in systemic inflammation, an increase in vagal activity [4], and enhancement of innate and acquired immune responses [5]. Moreover, the practice of physical exercise improves cardiorespiratory fitness, which, in turn, is closely related to the RHR.

Cardiorespiratory fitness is a direct measure of aerobic functional capacity [6,7], which allows global knowledge of the patient’s health status. Although the relationship between cardiorespiratory fitness and cancer mortality appears poorly studied, in recent years, interest in this relationship has increased. In fact, it is known that high cardiorespiratory fitness is a strong predictor of lower total cancer mortality [8]. Moreover, regarding cancer incidence, a recent meta-analysis concluded that in men, high cardiorespiratory fitness may be associated with a lower risk of colorectal and lung cancer [9]. Previous systematic review and meta-analysis showed that a reduced RHR is associated with high levels of cardiorespiratory fitness and with decreased risk of cancer and all-cause mortality [10], however, did not display site-specific cancer mortality results.

Since cardiorespiratory fitness is a solid predictor of all-cause morbidity and mortality in several diseases, including cancer, just as physical activity is [8,11,12], and because the RHR is an easy and feasible measurement to assess cardiorespiratory fitness, the aim of this systematic review and meta-analysis was to summarize the evidence available about the relationship between the RHR and site-specific risk of cancer and mortality.

## 2. Materials and Methods

This systematic review and meta-analysis was registered through the International Prospective Register of Systematic Reviews-PROSPERO (CRD42018095756). This meta-analysis was performed in accordance with the Preferred Reporting Items for Systematic Reviews and Meta-Analyses (PRISMA) [13] and Meta-Analysis of Observational Studies in Epidemiology (MOOSE) guidelines [14]. In addition, we followed the recommendations of the Cochrane Handbook for Systematic Reviews of Interventions [15]. 

### 2.1. Search Strategy

Studies were identified in the following databases from their inception to 24 September 2020: MEDLINE (via PubMed), Web of Science, EMBASE, and Cochrane Library. The search strategy included (“heart rate” OR “pulse” OR “resting heart rate” OR “beats”) AND (“cancer” OR “neoplasm” OR “carcinoma” OR “site-specific cancer” OR “cancer morbidity” OR “cancer mortality”) AND (“risk” OR “incide*” OR “cohort” OR “follow-up study” OR “prospective”). The literature search was complemented by scanning the reference list of published full-text articles and systematic reviews for relevant studies.

### 2.2. Selection Criteria

The systematic literature search was independently conducted by two reviewers (D.P.P.-C. and I.C.-R.), and disagreements were resolved by consensus; if necessary, a third researcher (V.M.-V.) was asked to evaluate the study in question. Reviewers were not blinded to authors, journals or institutions. The articles included were follow-up studies that analyzed the site-specific risk of cancer and/or cancer mortality associated with the RHR. The criteria for the exclusion of studies were as follows: (i) studies not written in English, French, Portuguese, or Spanish; (ii) studies not reporting the incidence of cancer or cancer mortality; (iii) studies not reporting the RHR; (iv) non-follow-up studies or studies that did not report empirical data (review articles, editorials, comments, guidelines, or case reports); (v) studies including patients with stablished cancer diagnosis at the beginning of the study; and (vi) duplicate reports of the same study. For the meta-analysis, when some studies had published multiple reports, we used the study that provided more detailed data and had the largest sample size.

### 2.3. Search and Data Extraction

Data extraction was performed independently by two authors (D.P.P.-C. and I.C.-R.) using a standardized data collection form. The following data were collected from each selected study: (i) name of the first author and year of publication; (ii) country; (iii) study name; (iv) period of data collection; (v) characteristics of the population: sample size, age mean, sex, race, and comorbidities; (vi) data concerning heart rate (test used and values) and cancer (type, incidence and/or mortality events); and (vii) main study exposures and outcomes. When some information was lacking, we emailed the corresponding author requesting the data.

### 2.4. Risk of Bias Assessment

We used the validated Newcastle-Ottawa scale for cohort studies to assess the quality of the included studies [16]. This scale assigns four points for quality of selection, two points for comparability, and three points for quality of outcome and adequacy of follow-up, with a maximal score of nine points. Since no explicit guidelines exist, according to these scores, the studies were rated as “good”, “fair”, or “poor” quality according to whether they scored six or more points, five points or less than five points, respectively, as done in previous studies [8,9]. 

### 2.5. Statistical Analysis

Due to the cut-offs used for RHR categories and the effect size measures in the studies included, we performed the following three strategies for dose-response analyses: (1) analysis of cancer mortality comparing the “less than 60 bpm” and “more than 60 bpm” categories; (2) analysis of cancer mortality comparing the “less than 60 bpm”, “60 to 80 bpm”, and “more than 80 bpm” categories; and (3) cancer mortality risk analysis for each 10–12 and 20 bpm increase in the RHR. The analysis strategy was established following these considerations:

When the studies provided odds ratios (ORs), these were converted to relative risks (RRs) to be combined in the meta-analysis [17]. For the meta-analyses of the risk of cancer mortality by categories of RHR levels, RRs were used as a risk measure. Furthermore, for analysis for different bpm increase (as reported by studies) in the RHR and risk of cancer mortality, the hazard ratio (HR) was used.

When studies presented several statistical risk-adjustment models, we considered the one including the largest number of additional covariates.

To calculate the mortality pooled risk estimates by RHR categories (“less than 60 bpm” and “more than 60 bpm”), we only considered studies providing the risk of cancer mortality compared to the same reference level of RHR. Nevertheless, to calculate the pooled estimate by three categories of bpm (<60 bpm, 60 to 80 bpm, and >80 bpm), we included studies that, while not reporting risk measures for exactly the same categories, did report risk estimate measures for RHR in the ranges that we have established for those three categories. 

When the studies provided the data as an increment of RHR (i.e., 10, 12, or 20 bpm), we calculated two pooled HR subgroups: one for studies that reported an increase of 10–12 bpm and another for studies that reported an increase of 20 bpm, both in overall and by sex.

The DerSimonian and Laird random effects method was used to compute pooled estimates of HRs or RRs and their respective 95% confidence intervals [95% confidence interval (CI)] for cancer mortality associated with RHR levels. The heterogeneity of results across studies was evaluated using the I2 statistic and was categorized as might not contain heterogeneity (0–40%), may represent moderate heterogeneity (30–60%), may represent substantial heterogeneity (50–90%), and may represent considerable heterogeneity (75–100%) [15]. In addition, the corresponding p values were considered.

Sensitivity analyses (systematic reanalysis while removing studies one at a time) were conducted to assess the robustness of the summary estimates. The results of the sensitivity analyses were considered meaningful when the resulting estimates were modified beyond the CIs of the original summary estimate.

Finally, publication bias was evaluated through visual inspection of funnel plots and by using the method proposed by Egger [18]. Statistical analyses were performed using StataSE software v16 (StataCorp, College Station, TX, USA).

## 3. Results

**Figure 1:**
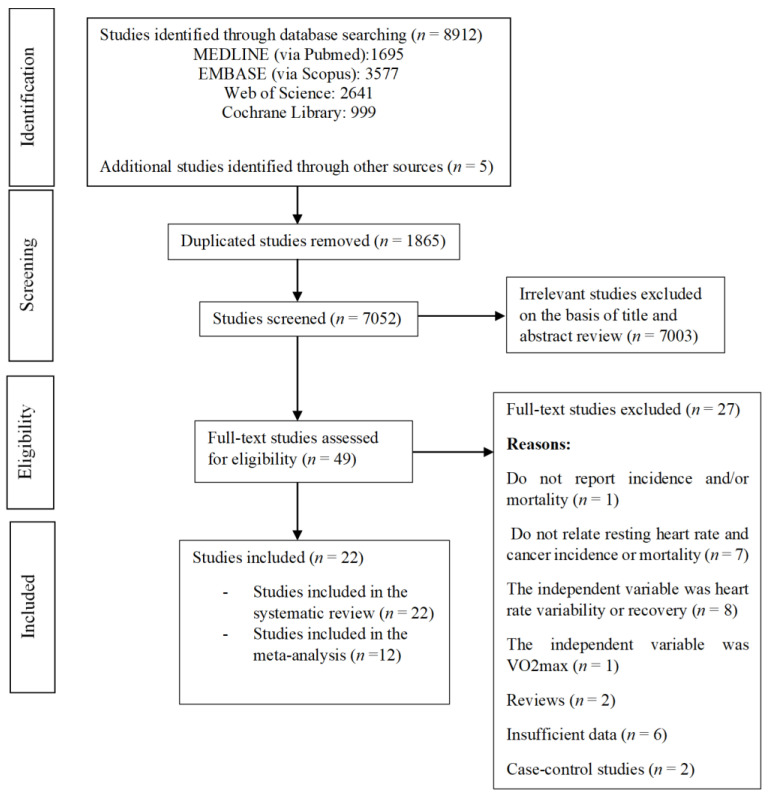
Literature search: Preferred Reporting Items for Systematic Reviews and Meta-Analyses (PRISMA) consort diagram.

Figure 1 depicts the flow of study selection. There were 8912 potentially eligible references retrieved in the electronic search, and five studies were identified through the list of references of other reviews and included studies. After removing duplicates, titles and abstracts were screened, and 49 were reviewed in full text to determine whether they clearly met the inclusion criteria. Finally, 22 studies were selected for the systematic review [19,20,21,22,23,24,25,26,27,28,29,30,31,32,33,34,35,36,37,38,39,40], and twelve of them were included in the meta-analysis [22,24,25,26,28,29,30,32,34,36,38,39] aimed to assess the relationship between RHR and cancer mortality (meta-analyses of cancer incidence could not be conducted because of the small number of studies and their heterogeneity; see results). The reasons that some full-text studies were excluded are summarized in Supplemental file S1.

The procedures used to measure the RHR were very varied: in four studies, RHR was measured manually [20,21,28,37], although two of them also included automated tools [37] or electrocardiogram (ECG) [21] measurements; twelve studies used ECG, and finally one study used sphygmomanometer and chronometer [39] and other automatic blood pressure monitor [32]. Five studies did not report the instruments used to measure the RHR [19,23,26,33,38].

Table 1 shows the characteristics of the studies and the participants. Studies were conducted in the USA, France, Germany, Puerto Rico, Finland, Israel, Australia, The Netherlands, and the United Kingdom. In total, the twelve studies included in the meta-analysis involved 752,899 participants, and the sample size of each individual study varied from 1233 to 498,103 participants aged from 18 to 95 years. The length of follow-up ranged from two to eight years.

### 3.1. Risk of Bias Assessment

All studies included in the meta-analysis received equal or more than six points based on the Newcastle-Ottawa scale, which indicates that the studies included were of high quality (Supplemental file S2).

### 3.2. Pooled Estimates

#### 3.2.1. Cancer Incidence

Among the 22 studies included in the systematic review, three studies reported all cancer incidences [20,33,35]. In addition, Table 2 displays the incidence of cancer by “type of cancer”, showing that six studies addressed the incidence of genitourinary cancer [20,21,31,33,35,37], four addressed the incidence of gastro-intestinal cancer [20,31,33,35], and three addressed the incidence of respiratory cancer [31,33,35]. Finally, three studies reported the incidence of “other cancers” such as breast, brain, or lymphoid cancer [32,33,35].

Unfortunately, a meta-analysis of data regarding cancer incidence was not possible because the studies reported these results using different reference categories of RHR levels.

#### 3.2.2. Cancer Mortality

Data about cancer mortality were analyzed using three different strategies: (i) we performed a meta-analysis including studies that reported cancer mortality by categories of mean RHR (≤60 bpm versus > 60 bpm); (ii) a second meta-analysis compared the risk of cancer mortality using three RHR categories (<60 bpm (reference level), 60–80 bpm, and >80 bpm); (iii) finally, an analysis was conducted assessing the effect of 10–12 and 20 bpm increase on cancer mortality risk.

- Cancer mortality comparing the “less than 60 bpm” and “more than 60 bpm” categories

**Figure 2:**
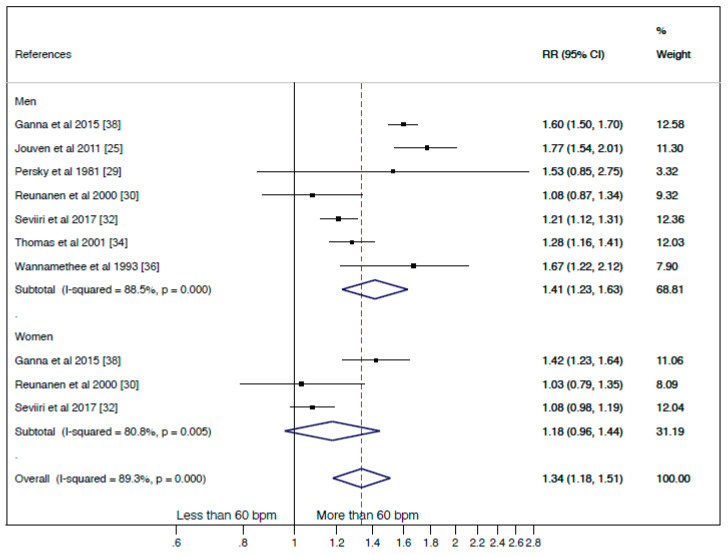
Forest plot of relative risk (RR) of cancer mortality for individuals with less than 60 beats per minute (bpm) of resting heart rate (RHR) category versus more than 60 bpm of RHR category, by gender and overall.

Seven prospective studies were included in this meta-analysis [25,29,30,32,34,36,38]; of them. The summary RR for “less than 60 bpm” vs. “more than 60 bpm” was 1.34 (95% CI: 1.18, 1.51; I2 = 89.3%, *p* < 0.001) (Figure 2). Evidence of publication bias was not found by funnel plot asymmetry and the Egger test for the cancer mortality estimate (*p* = 0.093). The pooled RR estimate was not significantly modified in magnitude or direction when individual study data were removed from the analysis one at a time (e.g., RRs of 1.24 to 1.42). 

A subgroup by gender was performed, even though only three studies included women in their sample. Figure 2 shows that RR estimates were 1.41 (95%CI: 1.23, 1.63; I2 = 88.5%, *p* = 0.000) and 1.18 (95% CI: 0.96, 1.44; I2 = 80.8%, *p* = 0.005) for men and women, respectively. The heterogeneity was not substantially modified when the gender subgroup analyses were performed.

Aimed to explore the high heterogeneity several subgroups analysis by type of measurement tool and type of cancer were attempted, but there were not enough studies in each group to perform them.

**Figure 3:**
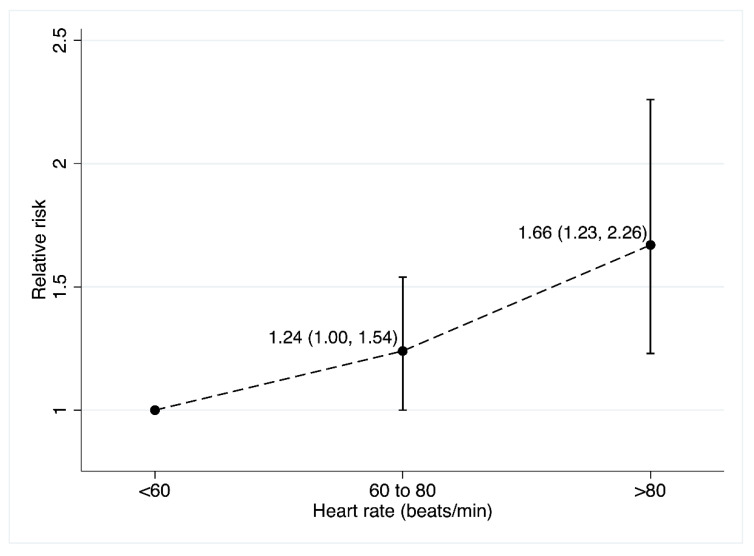
Relative risk (RR) of cancer mortality comparing the less than 60 beats per minute (bpm) category with 60 to 80 bpm and more 80 bpm categories.

Additionally, Figure 3 displays the cancer mortality by three RHR categories and shows that RR increases significantly when the RHR is above 80 bpm [RR: 1.66 (95% CI: 1.23, 2.26)] compared with when it is less than 60 bpm. However, when the 60 bpm category was compared with the 60 to 80 bpm group, the RR of cancer mortality was not significantly higher [RR: 1.24 (95% CI: 1.00, 1.54)].

- Analysis for 10–12 and 20 bpm increase and cancer mortality

**Figure 4:**
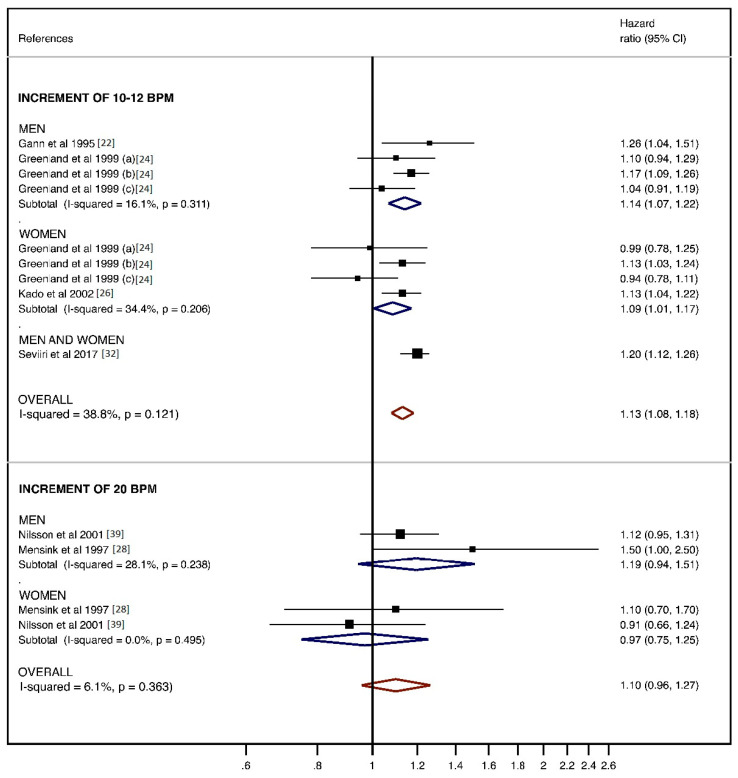
Analysis for 10–12 and 20 beats per minute (bpm) of resting heart rate (RHR) on risk of cancer mortality.

Six prospective studies reported data about cancer mortality by increments of RHR in bpm [22,24,26,28,32,39]. The overall HR for 10–12 bpm increase was 1.13 (95% CI: 1.08, 1.18; I2 = 38.8%, *p* = 0.121) and for 20 bpm increase was 1.10 (95% CI: 0.96, 1.27; I2 = 6.1%, P = 0.369). Subgroup analysis by sex showed pooled HRs for an increase of 10–12 bpm of 1.14 (95% CI: 1.07, 1.22; I2 = 16.1%, *p* = 0.311) for men and 1.09 (95% CI: 1.01, 1.17; I2 = 34.4%, *p* = 0.206) for women; and for an increase of 20 bpm of 1.19 (95% CI: 0.94, 1.51; I2 = 28.1%, *p* = 0.238) for men and 0.97 (95% CI: 0.75, 1.25; I2 = 0.0%, *p* = 0.495) for women (Figure 4). There was no evidence of publication bias by funnel plot asymmetry and Egger’s test for an increase of 10–12 bpm (*p* = 0.094). The pooled HR estimate was not significantly modified in magnitude or direction when individual study data were removed from the analysis one at a time (e.g., HRs of 1.12 to 1.15 for an increase of 10–12 bpm and HRs of 1.07 to 1.15 for an increase of 20 bpm).

## 4. Discussion

Our analyses show a positive association between RHR and the risk of cancer mortality, suggesting that a RHR less than 60 bpm seems to be a protective factor against the risk of cancer mortality. Similar result was found when comparing three RHR categories using less than 60 bpm as the reference category and, finally, in analysis estimating the effect of an increase of 10–12 bpm in the RHR on cancer mortality. Nevertheless, in these two last analyses, some inconsistencies can be observed, because while Figure 3 shows RHR > 80 bpm (RR = 1.66; CI: 1.23, 2.26), the analyses by increments of 10–12 or 20 bpm show lower effect sizes; this inconsistency in the RR or HR estimates could be attributed to differences in the number of studies included in each analysis. However, despite these differences, both results show the protective role of lower RHR against cancer mortality.

In a previous meta-analysis aimed at assessing the relationship between RHR, the risk of cardiovascular disease and cancer mortality provided similar estimates of increase in the risk of cancer mortality to our study [10], but it did not report analyses of cancer mortality risk by gender, bpm categories (less and more than 60 bpm; less than 60 bpm, 60 to 80 bpm, and more than 80 bpm) and bpm increments.

The mechanisms underlying the relationship between heart rate and cancer are complex and not entirely understood. It has been suggested that an elevated RHR indicates increased sympathetic activation and, consequently, an autonomic imbalance that has been associated with elevated risk not only of cardiovascular events but also of cancer and all-cause mortality [41]. Furthermore, this imbalance increases the stimulation of adrenergic activity, producing an increase in neurotrophic factors that stimulate the proliferation of epithelial cells, which could affect inflammation, angiogenesis, tissue invasion, the cellular immune response, and epithelial–mesenchymal transition [42]. Additionally, genetic influence has been claimed as a potential mechanism linking RHR and cancer [43]. Indeed, two possible approaches have been raised: (i) genotype variants exert an effect on mortality directly through the heart rate as a mediator, or (ii) these variants share the same underlying biology, resulting in increases in heart rate as well as the risk of mortality [44]. However, other potential approaches involve the basic metabolic rate, energy, and free radicals, causing general cumulative damage and affecting the life span [45]. 

In recent decades, there has been growing evidence showing a positive association between RHR and all-cause mortality [10,46]. However, the role of RHR in the healthy population and its impact on cancer mortality remain unclear. Among the several factors that could influence the RHR [47], one of the most important is physical exercise, since long-term aerobic exercise affects parasympathetic nerve activity by increasing the stroke volume and, as consequence, decreasing the RHR [48]. Additionally, in developed countries, individuals who are physically active usually tend to have healthier habits such a balanced diet, do not smoke, they are not overweight or obese, which are factors related with the risk of cancer [49]. Moreover, exercise improves cardiorespiratory fitness, which has been inversely associated with adiposity [50], and adiposity has been related to cancer incidence through hormonal mechanisms such as altered sex hormone metabolism, increased bioavailability of insulin-like growth factor I or adipokine pathophysiology. Moreover, new hypotheses have recently emerged, including microbiome effects [51]. 

Because genetic determinants of RHR could be involved in the origin of cancer [44] and because the RHR is associated with obesity, an intriguing question that arises is whether a high RHR is merely a marker of risk or an independent risk factor increasing the risk of developing at least some specific types of cancer. Nevertheless, the relationship between elevated RHR and higher risk of cancer mortality could be confounded by other related factors such as body mass index, physical activity, cardiorespiratory fitness [52].

Our study shows that a RHR lower than 60 bpm seems to be a protective factor against the risk of cancer mortality, nevertheless this analysis shows a high heterogeneity, which could be caused by the variability among studies in the strength of the association rather than the disparity in the effect´s direction. There exists the possibility that some part of heterogeneity may be due to clinical differences such as, age, duration of follow-up, type of cancer, or other confounders. When the subgroup analysis by gender was performed, a slight reduction in heterogeneity was shown (Figure 2 and Figure 4), nevertheless other subgroups analyses by type of cancer or by the instrument used to measure RHR were not possible because of the scarcity of studies. The duration of follow-up was very varied among the studies included in this analysis, and it could be also a source of heterogeneity. Of note, the only study that reported a negative relationship between RHR and risk of cancer mortality [30] had, along with the Jouven et al. study [25], the longest follow-up. A longer follow-up could imply patients with higher age and the possibility of more undiagnosed comorbidities affecting the RHR, however these two studies found results in opposite directions. Regarding the number of covariates as a potential source of heterogeneity, it should be noted that all studies included in the analysis comparing “less” and “more than 60 bpm” categories were adjusted by age and smoke habit, but in some studies such as the one by Jouven et al. [25] and the one by Ganna et al. [38], the estimates were not additionally adjusted by other important confounders such as body mass index or physical activity, which are also known to influence RHR [52] and it may be convenient to take them into account.

Another important recognized confounder is the cardiorespiratory fitness level. A previous meta-analysis concluded that moderate and high levels of cardiorespiratory fitness reduce the risk of lung, colorectal, and all cancer sites [9]. Lower RHR values have been associated with high physical fitness, even in patients with cancer [52]. In these patients, a previous meta-analysis revealed that heart rate variability is usually decreased, probably produced by the characteristic autonomic dysfunction related with cancer disease. In addition, heart rate variability is related with the clinical course of cancer, being a predictor of cancer patients’ prognosis [53]. Nevertheless, in cancer patients, there are many factors that could influence the values of RHR such as anticancer therapy (chemotherapy and chest radiation therapy), depression, and tobacco smoking habit [52]. Of these, chemotherapy is especially important due to the potential heart damage as well as others cardiovascular disorders including arrhythmias, ischemia, thrombo-embolism, and even heart failure [41,52].

Our findings suggest that a high RHR is a risk biomarker not only for cardiovascular disease and all-cause mortality [41,52,54] but also for cancer; in fact, the elevation of RHR could be consider a predictor for poor survival in cancer patients [52]. However, the measurement of RHR as a risk biomarker has not yet been recommended in clinical guidelines [55,56]. Some inconsistences in the results of studies relating RHR with the incidence of cancer [54] and the lack of agreement about the optimal cut off for both cardiovascular events and cancer could be behind this. Our data, as well as other meta-analyses relating RHR with cardiovascular events [10], suggest that a threshold of >80 bpm may not be an indicator of disease but a clinical sign of enough importance to encourage individuals to modify behaviors related to an increase in the RHR.

This meta-analysis has several limitations that should be highlighted. First, the studies used different RHR levels as reference values for their estimates (HR or RR), which limited the number of studies eligible for inclusion in the meta-analysis. Second, cancer risk is related to several lifestyle habits, such as smoking, physical activity, and a healthy diet [47]; thus, although the studies included adjustments for many of these risk factors, it would have been interesting to analyze the influence of these risk factors on the estimates of the relationship between RHR and cancer risk. Third, the RHR is sensitive to factors such as infections, recent physical activity, anxiety, stress, and use of medications, such as use of beta-blockers [57]. Fourth, the procedures used to measure RHR and the confounding variables for which associations were adjusted were different in the included studies, which could be a potential cause of bias and/or high heterogeneity. Fifth, although the studies included that the participants were healthy, they did not report whether the presence of cancer was ruled out, ensuring the absence of cancer at the time of RHR. Finally, the relationship between the covariate adjustment and the effect side was not possible to analyze due to the wide range of multivariable adjustment used between studies. Therefore, changes in the RHR, as an independent predictor of mortality, should still be cautiously considered.

## 5. Conclusions

In conclusion, our findings suggest that the RHR is associated with cancer mortality and could be related to the incidence of some types of cancer. Likewise, this study suggests the potential use of the RHR as a prognostic factor for cancer in clinical settings. Future studies are needed to verify the association of the RHR with cancer incidence and to determine feasible and efficient strategies to modify the RHR to improve cancer progression and overall survival.

The wide use of exercise trackers and personal heart rate monitors makes research in the field of heart rate important not only for practitioners but also for individuals. Because the clinical accuracy of cut-offs for use in clinical practice has not been fully assessed, an RHR < 80 bpm could be used as a positive prognostic indicator in cancer patients.

## Figures and Tables

**Table 1:** Characteristics of studies included in the systematic review.

Reference	Country	Study Name	Period of Data Collection/Follow-Up	Sample Size (Male/Female) Race (White/Black/Asian/Latin/Unknow) Type of Population	Age Distribution	RHR Assessment	Number of Cancer Deaths/Cancer Events	Main Study Exposures	Main Study Outcomes
Batty et al. 2010 [19]	UK	Original Whitehall Study	1967–1970 40 years	*1183M* Race NR General population	40–69	NR	Mortality: All cancer: 244	Walking pace, LTPA, and RHR	Disease-specific mortality
Cerhan et al. 1999 [20]	USA	Iowa 65+ Rural Health Study	1982–1993 8.5 years	*2819M&F* Race NR General population	65–102	Manually	Cancer events: All cancer: 231	RHR	Cancer incidence
Dekker et al. 2000 [40]	USA	ARIC Study	1987–1989 ~5 years	*15,792 M&F* Race NR General population	45–64	ECG	Mortality: All cancer:209	RHR and HRV	Cancer and cardiovascular mortality
Fitzpatrick et al. 2001 [21]	USA	The Cardiovascular Health Study (CHS)	1989–1990 1992–1993 5.6 years	*2442M* 318B/2124 Race NR General population	≥65	Manually and ECG	Cancer events: Prostate: 209	BP and RHR	Prostate cancer incidence
Friedman et al. 1996 [37]	USA	Kaiser Permanent Medical Care Program (KPMCP)	1964–1972 ~30 years	*58,704M* 46,145W/7535B/2534A/2490U General population	30–79	Manually, Air Shield and Godart automated device	Mortality: Prostate: 464 Cancer events: Prostate: 2297	BP and RHR	Prostate cancer mortality and incidence
Gann et al. 1995 [22]	USA	Chicago Heart Association Detection Project	1967–1973 19.2 years	*22,380M* 20,184W/1487B/709U General population	15–90	ECG	Mortality: Prostate: 464	RHR	Prostate cancer mortality
Ganna et al. 2015 [38]	UK	UK Biobank	2007–2010 4.9 years	*498,103 M&F* Race NR General population	37–73	NR	Mortality: All cancer: 5083	Several health-related variables. RHR	Cardiovascular, respiratory, digestive, cancer, and other causes mortality
Garcia-Palmieri et al. 1981 [23]	Puerto Rico	Puerto Rico Heart Health Program	1965–1969 ~8 years	*9824M* Latin General population	45–64	NR	Mortality: All cancer: 179	Serum cholesterol	Cancer mortality
Greenland et al. 1999 [24]	USA	Chicago Heart Association Detection Project	1967–1973 22 years	18,787M/12,994F 3357B/28,424 Race NR General population	18–74	ECG	Mortality: All cancer: 724	RHR	Cardiovascular and noncardiovascular mortality
Jouven et al. 2011 [25]	France	-	1967–1972 25 years	6101M Race NR General population	42–53	ECG	Mortality: All cancer: 771 Respiratory cancer: 241 Digestive cancer: 210	RHR	Cancer mortality
Kado et al. 2002 [26]	USA	Study of Osteoporotic Fractures	1986–1988 8.9 years	9704F White General population	≥65	NR	Mortality: All cancer: 580	RHR	Osteoporotic fractures and mortality
Kristal-Boneh et al. 2000 [27]	Israel	CORDIS Study	1985–1987 8 years	3527M White General population	43	ECG	Mortality: All cancer: 45	RHR	Cardiovascular, cancer, and all-cause mortality
Mensink et al. 1997 [28]	Germany	Spandau Health Test	1984–1991 12 years	1798M/2908F Race NR General population	40–80	Manually	Mortality: All cancer: 126	RHR	Cardiovascular, cancer, and all-cause mortality
Nilsson et al. 2001 [39]	Sweden	Malmö Preventive Project	1974–1992 17 (*M*)/12 (*F*) years	13,466M/9467F Race NR General population	46.7	Sphygmomanometer and chronometer	Mortality: All cancer: 841	Sleep deprivation and RHR	Cardiovascular, cancer, and all-cause mortality
Perskly et al. 1981 [29]	USA	Chicago Peoples Gas Company Study Chicago Western Electric Company Study Chicago Heart Association Detection Project	1958–1976 1957–1974 1967–1979 18 years	1233M White General population 1899M White General population 5784 White General population	40–59 40–55 45–64	ECG	Mortality: All cancer: 99 Mortality: All cancer: 78 Mortality: All cancer: 95	RHR	Cancer mortality
Reunanen et al. 2000 [30]	Finland	-	1966–1972 23 years	5598M/5119F Race NR General population	30–59	ECG	Mortality: All cancer: 364	RHR	Cardiovascular, cancer, and all-cause mortality
Severson et al. 1989 [31]	USA	-	1965–1968 20 years	8006M Asian General population	46–65	ECG	Cancer events: Colon: 193 Rectum: 95 Stomach: 171 Lung: 194 Prostate: 206 Bladder: 70	Physical activity	Cancer incidence
Seviiri et al. 2017 [32]	Australia	Melbourne Collaborative Study	1990–1994 21.9 years	17,045M/24,469F Race NR General population	40–69	Automatic blood pressure monitor	Mortality: All cancer: 3618 Bladder: 69 Brain: 124 Breast: 277 Colorectal: 460 Kidney: 73 Lung: 566 Lymphoid: 415 Prostate: 247 Ovarian: 122 Skin: 122 Upper aerodigestive tract: 118 Other cancer: 1025	RHR	Cardiovascular, cancer, and all-cause mortality
Steenland et al. 1995 [33]	USA	NHANES 1	1971–1975 15 years	13,054M&F 11,096W/1958 Race NR General population	25–74	NR	Cancer events: All cancer: 1335 Lung: 210 Colorectal: 176 Breast: 163 Prostate: 156 Bladder: 56 Pancreas: 54 Leukemia: 44 Kidney: 41 Skin: 41 Stomach: 33 Ovary: 27 Liver: 25 Brain: 21 Cervix: Uterus: 43 Esophagus: 17 Larynx: 15 Melanoma: 10 Thyroid: 5 Oral: 21 Other hematopoietic: 63 Other: 94	Diabetes, cholesterol, RHR, and physical activity	Cancer incidence
Thomas et al. 2001 [34]	France	-	1970–1978 8 years	125,513M Race NR General population	20–95	ECG	Mortality: All cancer: 3618 Respiratory cancer: 416 Digestive cancer: 347 Genito-urinary cancer: 113	BP and RHR	Cancer mortality
van Kruijsdijk et al. 2014 [35]	Netherlands	Second Manifestations of ARTerial disease study (SMART)	1996–2012 ~ 6 years	4433M/1574F 5707W/300 Race NR Vascular disease patients	18–80	ECG	Mortality: All cancer: 56 Cancer events: All cancer: 126 Lung: 27 Colorectal: 17 Breast: 4 Prostate: 24	RHR	Cancer mortality and incidence, and all-cause mortality
Wannamethee et al. 1993 [36]	UK	British Regional Heart Study	1978–1980 9.5 years	7735M Race NR General population	40–59	ECG	Mortality: All cancer: 217	RHR and physical activity	Cancer and other noncardiovascular mortality

RHR: resting heart rate; UK: United Kingdom; NR: No reported; LTPA: Leisure time physical activity; USA: United State of America; ECG: electrocardiogram; BP: blood pressure.

**Table 2:** Prospective cohort studies included in meta-analyses of resting heart rate and cancer mortality.

Reference	Association Measurement	RHR Level Comparison	Mortality	Cancer Events	Adjustment Variables
**ALL CANCER**					
Batty et al. 2010 [19]	HR	≤64 beats/min 65–74 beats/min ≥75 beats/min	1.00 1.10 (0.81, 1.49) 1.08 (0.79, 1.49)	NR	Age, employment grade, BMI, smoking and forced expiratory volume in 1 s
Cerhan et al. 1999 [20]	HR	<63 beats/min 63–68 beats/min 69–74 beats/min 75–82 beats/min >82 beats/min	NR	1.00 **1.68 (1.06, 2.66)** 1.54 (0.95, 2.49) **1.62 (1.03, 2.55)** **1.66 (1.03, 2.65)**	Age, BMI, smoking, and physical activity
*Men*					
*Women*				1.00 1.06 (0.71,1.57) 0.84 (0.55, 1.30) 1.03 (0.70, 1.50) 1.15 (0.77, 1.73)	
Dekker et al. 2000 [40]	RR	≤63 beats/min 64–71 beats/min ≥72 beats/min	1.00 1.22 (0.79–2.38) 1.04 (0.62–1.76) 1.61 (1.05–2.48) 1.39 (0.86–2.25)	NR	Age, sex, race, field center, current smoking, and cigarette-years.
Ganna et al. 2015 [38] *Men*	RR	30.5–60 beats/min 60–66 beats/min 66–71.5 beats/min 71.5–79 beats/min 79–174 beats/min	1.00 1.2 (1.1, 1.4) 1.4 (1.2, 1.6) 1.6 (1.4, 1.8) 2.2 (2.0, 2.5) 0.7 (0.6,0.8) 0.8 (0.7, 0.9) 0.9 (0.8, 1.0) 1.0 1.4 (1.3–1.6)	NR	Age
*Women*					
Greenland et al. 1999 [24]	HR	Increment of 12 beats/min		NR	Age, education, serum cholesterol, smoking, BMI, major and minor electrocardiographic abnormalities, race, diabetes, and SBP.
*Men aged 18*–*39 years*			1.10 (0.94, 1.29)		
*Women aged 18–39 years*			0.99 (0.78, 1.25)		
*Men aged 40–59 years*			**1.17 (1.09, 1.26)**		
*Women aged 40*–*59 years*			**1.13 (1.03, 1.24)**		
*Men aged 60*–*74 years*			1.04 (0.91, 1.19)		
*Women aged 60*–*74 years*			0.94 (0.78, 1.11)		
Jouven et al. 2011 [25]	RR	<60 beats/min 60–67 beats/min 68–73 beats/min >73 beats/min	1.00 **1.60 (1.20, 2.00)** **1.60(1,30, 2.00)** **2.40 (1.90, 2.90)**	NR	Age and smoking
Kado et al. 2002 [26]	HR	Increment of 10 beats/min	**1.13 (1.04, 1.22)**	NR	Age, weight, self-reported health, physical activity, hyperthyroidism, hypertension, diabetes, and smoking.
		<80 beats/min ≥80 beats/min	1.00 1.20 (0.90, 1.50)		
Kristal-Boneh et al. 2000 [27]	HR	<70 beats/min 70–79 beats/min 80–89 beats/min ≥90 beats/min	1.00 0.86 (0.30, 2.00) 1.55 (0.70, 3.50) 1.13 (0.40, 3.00)	NR	Age, smoking, education, sport, and hemoglobin
Mensink et al. 1997 [28]	HR	Increment of 20 beats/min	1.50 (1.00, 2.50)	NR	Age, serum cholesterol, BMI, SBP, smoking and diabetes
*Men*					
*Women*			1.10 (0.70, 1.70)		
Nilsson et al. 2001 [39] *Men*	HR	Increment of 20 beats/min	1.12 (0.95, 1.31) 0.91 (0.66, 1.24)	NR	Age, serum cholesterol, BMI, SBP, smoking, and alcohol problematic drinking habits.
*Women*					

Perskly et al. 1981 [29]	RR	≤60 beats/min 61–67 beats/min 68–74 beats/min 75–79 beats/min ≥80 beats/min	1.00 1.2 (0.55, 2.61) 1.38 (0.69, 2.76) 1.54 (0.79, 3.01) **2.54 (1.34, 4.82)**	NR	Age, SBP, serum cholesterol, relative weight, and smoking
Reunanen et al. 2000 [30]	RR	≤60 beats/min 61–83 beats/min ≥84 beats/min	1.00 0.90 (0.69, 1.18) 0.89 (0.64, 1.24)	NR	Age, smoking, blood pressure, serum cholesterol, diabetes, body mass index, perceived health, job, and leisure time physical activity
Seviiri et al. 2017 [32]	HR	Increment of 10 beats/min	**1.10 (1.06, 1.13)**	NR	Age, sex, country of birth, level of education, waist circumference, alcohol consumption, smoking, physical activity score, Alternative Healthy Eating Index, total serum cholesterol, sodium/potassium ratio, caffeine, blood pressure, and history of hypertension, angina, asthma, and diabetes
		<60 beats/min 60–69 beats/min 70–79 beats/min 80–89 beats/min ≥90 beats/min	1.00 1.08 (0.98, 1.19) **1.15 (1.04, 1.28)** **1.41 (1.24, 1.60)** **1.40 (1.16, 1.69)**		
Steenland et al. 1995 [33]	OR	<73 beats/min 73–79 beats/min 80–87 beats/min ≥88 beats/min	NR	1.00 0.97 (0.76, 1.24) 1.09 (0.86, 1.39) 0.97 (0.76, 1.22)	Age, BMI, smoking, alcohol, income, recreational physical activity
Thomas et al. 2001 [34]	RR	<60 beats/min 60–80 beats/min >80 beats/min	1.00 1.18 (0.98, 1.43) 1.33 (1.19, 1.49)	NR	Age, body mass index, gamma-Gt, tobacco, cholesterol, PP, triglycerides, and physical activity
van Kruijsdijk et al. 2014 [35]	HR	≤55 beats/min 56–62 beats/min 63–71 beats/min ≥72 beats/min	1.00 0.99 (0.68, 1.43) 1.18 (0.83, 1.69) 1.06 (0.73, 1.54)	1.00 0.90 (0.70, 1.16) 0.94 (0.73, 1.22) 1.03 (0.79, 1.34)	Age, sex, smoking, hemoglobin levels, beta-blockers, calcium channel-blockers, alpha-blockers, diuretics, BMI, diabetes mellitus, physical activity, and high sensitivity C-reactive protein
Wannamethee et al. 1993 [36]	RR	<60 beats/min 60–69 beats/min 70–79 beats/min 80–89 beats/min ≥90 beats/min	1.00 1.50 (0.92, 2.41) **1.67 (1.02, 2.66)** **2.08 (1.23, 3.49)** 1.65 (0.88, 3.03)	NR	Age, social class, smoking, body mass index, heavy alcohol drinking, systolic blood pressure, blood cholesterol, preexisting ischemic heart disease, physical activity, and forced expiratory volume in 1 s.
**GENITO-URINARY CANCER**					
Cerhan et al. 1999 [20]	HR	<63 beats/min 63–68 beats/min 69–74 beats/min 75–82 beats/min >82 beats/min	NR	1.00 **3.47 (1.29, 9.31)** 1.98 (0.66, 5.94) 1.98 (0.70, 5.62) **3.16 (1.15, 8.71)**	Age, BMI, smoking, and physical activity
**Prostate**					
Fitzpatrick et al. 2001 [21]	HR	<60 beats/min 60–69 beats/min 70–79 beats/min ≥80 beats/min	NR	1.00 1.20 (0.80, 1.70) 1.10 (0.70, 1.70) **1.60 (1.03, 2.50)**	Age, race and BMI
**Prostate**					
Friedman et al. 1996 [37]	RR	≤66 beats/min 67–74 beats/min 75–83 beats/min ≥84 beats/min	NR	1.00 0.96 (0.85, 1.09) 0.90 (0.79, 1.02) 0.89 (0.79, 1.01)	Age
**Prostate**					
Gann et al. 1995 [22]	RR	Increment of 10 beats/min	**1.26 (1.04, 1.51)**	NR	Age, BMI, serum cholesterol, SBP, smoking, postload plasma glucose, and education
**Prostate**					
		<63 beats/min 63–72 beats/min 73–78 beats/min 79–87 beats/min >87 beats/min	1.00 1.55 (0.69, 3.45) 1.85 (0.84, 4.08) **2.18 (1.01, 4.70)** **2.69 (1.28, 5.66)**		Age
Severson et al. 1989 [31] **Prostate** ****Bladder****	RR	≤71 beats/min 72–81 beats/min ≥82 beats/min	NR	1.00 1.12 (0.80, 1.55) 0.97 (0.69, 1.36) 1.00 1.01 (0.55, 1.87) 1.22 (0.69, 2.18)	Age and BMI Age, BMI and smoking
Seviiri et al. 2017 [32]	HR	Increment of 10 beats/min	1.07 (0.94, 1.20)	NR	Age, sex, country of birth, level of education, waist circumference, alcohol consumption, smoking, physical activity score, Alternative Healthy Eating Index, total serum cholesterol, sodium/potassium ratio, caffeine, blood pressure, and history of hypertension, angina, asthma, and diabetes
**Prostate**					
**Bladder**			1.05 (0.83, 1.33)		
**Kidney**			1.27 (1.03, 1.57)		
**Ovarian**			0.87 (0.71, 1.06)		
Steenland et al. 1995 [33]	OR	<73 beats/min 73–79 beats/min 80–87 beats/min ≥88 beats/min	NR	1.00 0.93 (0.59, 1.45) 1.20 (0.77, 1.87) 1.28 (0.83, 1.97)	Age, BMI, smoking. alcohol, income, recreational physical activity
Thomas et al. 2001 [34]	RR	<60 beats/min 60–80 beats/min >80 beats/min	1.00 1.23 (0.70, 2.17) 0.86 (0.58, 1.28)	NR	Age, BMI, gamma-Gt, tobacco, cholesterol, PP, triglycerides, and physical activity
van Kruijsdijk et al. 2014 [35]	HR	≤55 beats/min 56–62 beats/min 63–71 beats/min ≥72 beats/min	NR	1.00 0.85 (0.46, 1.56) **0.45 (0.21, 0.98)** 0.87 (0.45, 1.70)	Age, sex, smoking, hemoglobin levels, beta-blockers, calcium channel-blockers, alpha-blockers, diuretics, BMI, diabetes mellitus, physical activity, and high sensitivity C-reactive protein
**Prostate**					
**GASTRO-INTESTINAL CANCER**					
Cerhan et al. 1999 [20]	HR	<63 beats/min 63–68 beats/min 69–74 beats/min 75–82 beats/min >82 beats/min	NR	1.00 1.49 (0.35, 6.29) **3.94 (1.11, 14.05)** 3.10 (0.86, 11.17) 1.70 (0.40, 7.12)	Age, BMI, smoking, and physical activity
**Colorectal**					
Jouven et al. 2011 [25]	RR	<60 beats/min 60–67 beats/min 68–73 beats/min >73 beats/min	1.00 1.60 (1.00, 2.50) **1.60 (1,10, 2.50)** **2.30 (1.50, 3.30)**	NR	Age and smoking
Severson et al. 1989 [31] **Colon** **Rectum** **Stomach**	RR	≤71 beats/min 72–81 beats/min ≥82 beats/min	NR		
				1.00 **0.56 (0.39, 0.80)** **0.71 (0.51, 0.99)**	Age and BMI
				1.00 1.31 (0.78, 2.20) 1.41 (0.84, 2.36)	
				1.00 1.07 (0.73, 1.59) 1.34 (0.92, 1.95)	Age, BMI and smoking
Seviiri et al. 2017 [32] **Colorectal** **UADT**	HR	Increment of 10 beats/min	**1.18 (1.08, 1.29)** 1.16 (0.98, 1.38)	NR	Age, sex, country of birth, level of education, waist circumference, alcohol consumption, smoking, physical activity score, Alternative Healthy Eating Index, total serum cholesterol, sodium/potassium ratio, caffeine, blood pressure, and history of hypertension, angina, asthma, and diabetes
Steenland et al. 1995 [33]	OR	<73 beats/min 73–79 beats/min 80–87 beats/min ≥88 beats/min	NR	1.00 1.34 (0.76, 2.34) 1.08 (0.59, 1.98) 1.33 (0.75, 2.37)	Age, BMI, smoking. alcohol, income, recreational physical activity
**Colorectal**					
Thomas et al. 2001 [34]	RR	<60 beats/min 60–80 beats/min >80 beats/min	1.00 1.00 (0.72, 1.38) **1.23 (1.03, 1.57)**	NR	Age, BMI, gamma-Gt, tobacco, cholesterol, PP, triglycerides, and physical activity
van Kruijsdijk et al. 2014 [35]	HR	≤55 beats/min 56-62 beats/min 63–71 beats/min ≥72 beats/min	NR	1.00 0.71 (0.32, 1.53) 0.87 (0.41, 1.87) 1.82 (0.86, 3.84)	Age, sex, smoking, hemoglobin levels, beta-blockers, calcium channel-blockers, alpha-blockers, diuretics, BMI, diabetes mellitus, physical activity, and high sensitivity C-reactive protein
**Colorectal**					
**RESPIRATORY CANCER**					
Jouven et al. 2011 [25]	RR	<60 beats/min 60–67 beats/min 68–73 beats/min >73 beats/min	1.00 **1.80 (1.20, 2.70)** 1.50 (1,00, 2.30) **3.00 (2.10, 4.50)**	NR	Age and smoking
Severson et al. 1989 [31] **Lung**	RR	≤71 beats/min 72–81 beats/min ≥82 beats/min	NR	1.00 **1.06 (0.76, 1.48)** 0.70 (0.48, 1.01)	Age, BMI, and smoking
Seviiri et al. 2017 [32] **Lung**	HR	Increment of 10 beats/min	**1.19 (1.10, 1.29)**	NR	Age, sex, country of birth, level of education, waist circumference, alcohol consumption, smoking, physical activity score, Alternative Healthy Eating Index, total serum cholesterol, sodium/potassium ratio, caffeine, blood pressure, and history of hypertension, angina, asthma, and diabetes
Steenland et al. 1995 [33] **Lung**	OR	<73 beats/min 73–79 beats/min 80–87 beats/min ≥88 beats/min	NR	1.00 0.67 (0.42, 1.09) 0.91 (0.57, 1.45) 1.21 (0.79, 1.84)	Age. BMI, smoking. alcohol, income, recreational physical activity
Thomas et al. 2001 [34]	RR	<60 beats/min 60–80 beats/min >80 beats/min	1.00 1.25 (0.88, 1.77) **1.52 (1.25, 1.85)**	NR	Age, BMI, gamma-Gt, tobacco, cholesterol, PP, triglycerides, and physical activity
van Kruijsdijk et al. 2014 [35] **Lung**	HR	≤55 beats/min 56–62 beats/min 63–71 beats/min ≥72 beats/min	NR	1.00 0.80 (0.46, 1.40) 0.87 (0.51, 1.50) 0.86 (0.50, 1.48)	Age, sex, smoking, hemoglobin levels, beta-blockers, calcium channel-blockers, alpha-blockers, diuretics, BMI, diabetes mellitus, physical activity, and high sensitivity C-reactive protein
**OTHER CANCERS**					
Seviiri et al. 2017 [32]	HR	Increment of 10 beats/min		NR	Age, sex, country of birth, level of education, waist circumference, alcohol consumption, smoking, physical activity score, Alternative Healthy Eating Index, total serum cholesterol, sodium/potassium ratio, caffeine, blood pressure, and history of hypertension, angina, asthma, and diabetes
**Breast**			**1.15 (1.02, 1.30)**		
**Brain**			0.91 (0.75, 1.09)		
**Lymphoid**			1.05 (0.95, 1.15)		
Steenland et al. 1995 [33]	OR	<73 beats/min 73–79 beats/min 80–87 beats/min ≥88 beats/min	NR	1.00 0.85 (0.54, 1.34) 0.86 (0.55, 1.35) 0.81 (0.52, 1.26)	Age. BMI, smoking. alcohol, income, recreational physical activity
**Breast**					
van Kruijsdijk et al. 2014 [35]	HR	≤55 beats/min 56–62 beats/min 63–71 beats/min ≥72 beats/min	NR	1.00 1.40 (0.40, 4.83) 0.92 (0.25, 3.40) 0.54 (0.13, 2.21)	Age, sex, smoking, hemoglobin levels, beta-blockers, calcium channel-blockers, alpha-blockers, diuretics, BMI, diabetes mellitus, physical activity, and high sensitivity C-reactive protein
**Breast**					

Bold values indicate *p* < 0.005. RHR: resting heart rate; HR: hazard ratio; BMI: body mass index; RR: relative risk; SBP: systolic blood pressure; RR: relative risk; OR: odds ratio; NR: Not reported. The relation of major sub-type cancer mortality RR or HR with RHR is available graphically in Supplemental file S3.

## Data Availability

No new data were created or analyzed in this study. Data sharing is not applicable to this article.

## Supplementary Materials

The following are available online at https://www.mdpi.com/2077-0383/10/7/1354/s1, Supplemental file S1: Excluded articles and reasons; Supplemental file S2: Newcastle-Ottawa Scale used to assess the risk of bias; Supplemental file S3: Relationship between major sub-type cancer mortality and resting heart rate (more information in Table 2).
