# Supplementary material for: Resting Heart Rate as a Predictor of Cancer Mortality: A Systematic Review and Meta-Analysis
Source: J Clin Med. 2021 Mar 25;10(7):1354. doi: 10.3390/jcm10071354 (PMC8037294; doi:10.3390/jcm10071354)

Supplemental file S1: Excluded articles and reasons

Do not report incidence and/or mortality:

1. Burnett D, Kluding P, Porter C, Fabian C, Klemp J. Cardiorespiratory fitness in breast cancer survivors. SpringerPlus. 2013;2(1):1-7.

Do not relate resting heart rate and cancer incidence or mortality:

2. Byun YH, Kim SY, Mok Y, Kim Y, Jee SH. Heart Rate Recovery and Cancer Risk: Prospective Cohort Study. Asia Pac J Public Health. 2018;30(1):45-55.
3. Fujiura Y, Adachi H, Tsuruta M, Jacobs Jr DR, Hirai Y, Imaizumi T. Heart rate and mortality in a Japanese general population: An 18-year follow-up study. Journal of Clinical Epidemiology. 2001;54(5):495-500.
4. Wang A, Chen S, Wang C, Zhou Y, Wu Y, Xing A, et al. Resting Heart Rate and Risk of Cardiovascular Diseases and All-Cause Death: The Kailuan Study. Plos One. 2014;9(10).
5. Theobald H, Wandell PE. Effect of heart rate on long-term mortality among men and women. Acta Cardiologica. 2007;62(3):275-9.
6. Saxena A, Minton D, Lee D-c, Sui X, Fayad R, Lavie CJ, et al. Protective Role of Resting Heart Rate on All-Cause and Cardiovascular Disease Mortality. Mayo Clinic Proceedings. 2013;88(12):1420-6.
7. Park J, Kim JH, Park Y, Park SJ, Cheon JH, Kim WH, et al. Resting heart rate is an independent predictor of advanced colorectal adenoma recurrence. Plos One. 2018;13(3).
8. Anker MS, Frey MK, Goliasch G, Bartko PE, Prausmüller S, Gisslinger H, et al. Increased resting heart rate and prognosis in treatment-naïve unselected cancer patients: results from a prospective observational study. European Journal of Heart Failure. 2020;22(7):1230-1238.

The independent variable was heart rate variability or recovery:

9. Chiang J-K, Kuo TBJ, Fu C-H, Koo M. Predicting 7-Day Survival Using Heart Rate Variability in Hospice Patients with Non-Lung Cancers. Plos One. 2013;8(7).

10. Ha D, Stephans K, Choi H, Zell K, Wang X-F, Minai OA, et al. Heart rate recovery and survival in patients undergoing stereotactic body radiotherapy for treatment of early-stage lung cancer. Journal of Radiosurgery and Sbrt. 2015;3(3):193-201.
11. Ha D, Malhotra A, Ries AL, O'Neal WT, Fuster MM. Heart rate variability and heart rate recovery in lung cancer survivors eligible for long-term cure. Respiratory Physiology & Neurobiology. 2019;269.
12. Hu S, Lou J, Zhang Y, Chen P. Low heart rate variability relates to the progression of gastric cancer. World Journal of Surgical Oncology. 2018;16(1).
13. Kloter E, Barrueto K, Klein SD, Scholkmann F, Wolf U. Heart Rate Variability as a Prognostic Factor for Cancer Survival - A Systematic Review. Frontiers in Physiology. 2018;9.
14. Niederer D, Vogt L, Thiel C, Schmidt K, Bernhoerster M, Lungwitz A, et al. Exercise Effects on HRV in Cancer Patients. International Journal of Sports Medicine. 2013;34(1):68-73.
15. Wang Y-M, Wu H-T, Huang E-Y, Kou YR, Hseu S-S. Heart Rate Variability Is Associated with Survival in Patients with Brain Metastasis: A Preliminary Report. Biomed Research International. 2013.
16. Shukla RS, Aggarwal Y. Time-domain heart rate variability-based computer-aided prognosis of lung cancer. Indian Journal of Cancer. 2018;55(1):61-5.

The independent variable was VO2max:

17. Laukkanen JA, Pukkala E, Rauramaa R, Makikallio TH, Toriola AT, Kurl S. Cardiorespiratory fitness, lifestyle factors and cancer risk and mortality in Finnish men. Eur J Cancer. 2010;46(2):355-63.

Reviews:

18. Mensink GBM, Hoffmeister H. Correlating resting heart rate with mortality risk. Cardiology Review. 1998;15(5):13-6.
19. Aune D, Sen A, o'Hartaigh B, Janszky I, Romundstad PR, Tonstad S, et al. Resting heart rate and the risk of cardiovascular disease, total cancer, and all-cause mortality - A systematic review and dose-response meta-analysis of prospective studies. Nutr Metab Cardiovasc Dis. 2017;27(6):504-17.

Insufficient data:

20. Palmier J, Lanzrath BJ. Laboratory and biometric predictors of cancer-related mortality in an insured population. Journal of insurance medicine (New York, NY). 2012;43(3):162-8.

21. Alhalabi L, Singleton MJ, Oseni AO, Shah AJ, Zhang ZM, Soliman EZ. Relation of Higher Resting Heart Rate to Risk of Cardiovascular Versus Noncardiovascular Death. Am J Cardiol. 2017;119(7):1003-7.

22. Bohm M. Heart rate: from heart failure to chronic diseases and cancer. Is there a role for supportive care by heart rate reduction? Eur J Heart Fail. 2017;19(2):250-2.

23. Zhang D, Wang W, Li F. Association between resting heart rate and coronary artery disease, stroke, sudden death and noncardiovascular diseases: a meta-analysis. Cmaj. 2016;188(15):E384-e92.

24. Anker MS, Ebner N, Hildebrandt B, et al. Resting heart rate is an independent predictor of death in patients with colorectal, pancreatic, and non-small cell lung cancer: results of a prospective cardiovascular long-term study. European journal of heart failure 2017; 18 (12): 1524-1534.

25. Wang Y, Chen S, Zhang Y, Emstsen L, Lavie CJ, Hooker SP, Chen Y, Sui X. Nonexercise Estimated Cardiorespiratory Fitness and All-Cancer Mortality: the NHANES III Study. Mayo Clin Proc 2018; 93(7):848-856.

Cases-control studies

26. Kwon YJ, Lee HS, Cho MR, Kim SN, Jeon JY, Kim NK, Lee JW. Association between resting heart rate and colorectal cancer: Results from a case-controlled study. Int J Environ Res Public Health. 2019 Aug 12;16(16). pii: E2883.

27. Anker MS, Ebner N, Hildebrandt B, Springer J, Sinn M, Riess H, Anker SD, Landmesser U, Haverkamp W, Haehling S. Resting heart rate is an independent predictor of death in patients with colorectal, pancreatic, and non-small cell lung cancer: results of a prospective cardiovascular long-term study. European Journal of Heart Failure. 2016; 18:1524-1534.

Supplemental file S2. Newcastle-Ottawa Scale used to assess the risk of bias.

		**Greenland et 1999**	**Mensink et al 1997**	**Nilsson et al 2001**	**Kado et al 2002**	**Seviiri et al 2017**	**Persky et al 1981**	**Ganna et al 2015**	**Reunanen et al 2000**	**Thomas et al 2001**	**Wannamethee et al 1993**	**Jouven et al 2011**
**Selection**	Representativeness of the exposed cohort	★		★		★		★	★			
	Selection of the non-exposed	★	★	★	★	★	★	★	★	★	★	★
	Ascertainment of exposure	★	★	★		★	★		★	★	★	★
	Outcome of interest not present at start	★	★	★	★	★	★	★	★	★	★	★
**Comparability**	Comparability: age and sex	★	★	★	★	★	★	★	★	★	★	★
	Comparability: additional factors	★	★	★	★	★	★	★	★	★	★	★
**Outcome**	Assessment of outcome	★	★	★	★	★		★	★	★	★	★
	Follow-up long enough	★	★	★		★	★		★	★	★	★
	Adequacy of follow-up	★		★	★	★			★			★

One star can be assign to the "representativeness" item whether the exposed cohort is truly or somewhat representative of the community. In the "selection of the non-exposed" item, one star will be assign whether the non-exposed cohort was drawn from the same community as the exposed cohort. One start can be assign to the "ascertainment of exposure" whether it was accomplished by secure record or strutured interview. In addition, one star will be assign whether the outcome of interest was not present at the start of the study. For comparability, two stars could be assign whether the exposure of interest was adjusted by age, sex, and additional factors. For the "assessment of outcome" one start will be assign whether independent or blind assessment, secure records or record linkage were stated in the paper. For the last two items, "follow-up long enough" and "adequacy of follow-up" one start can be assign for each, whether an acceptable follow-up long enough for the outcomes to occur and whether the follow-up ensure that losses were not related to the exposure or the outcome. Empty cells mean that the studies do not comply previous conditions.

Supplemental file S3. Relationship between major sub-type cancer mortality and resting heart rate (more information in table 2).

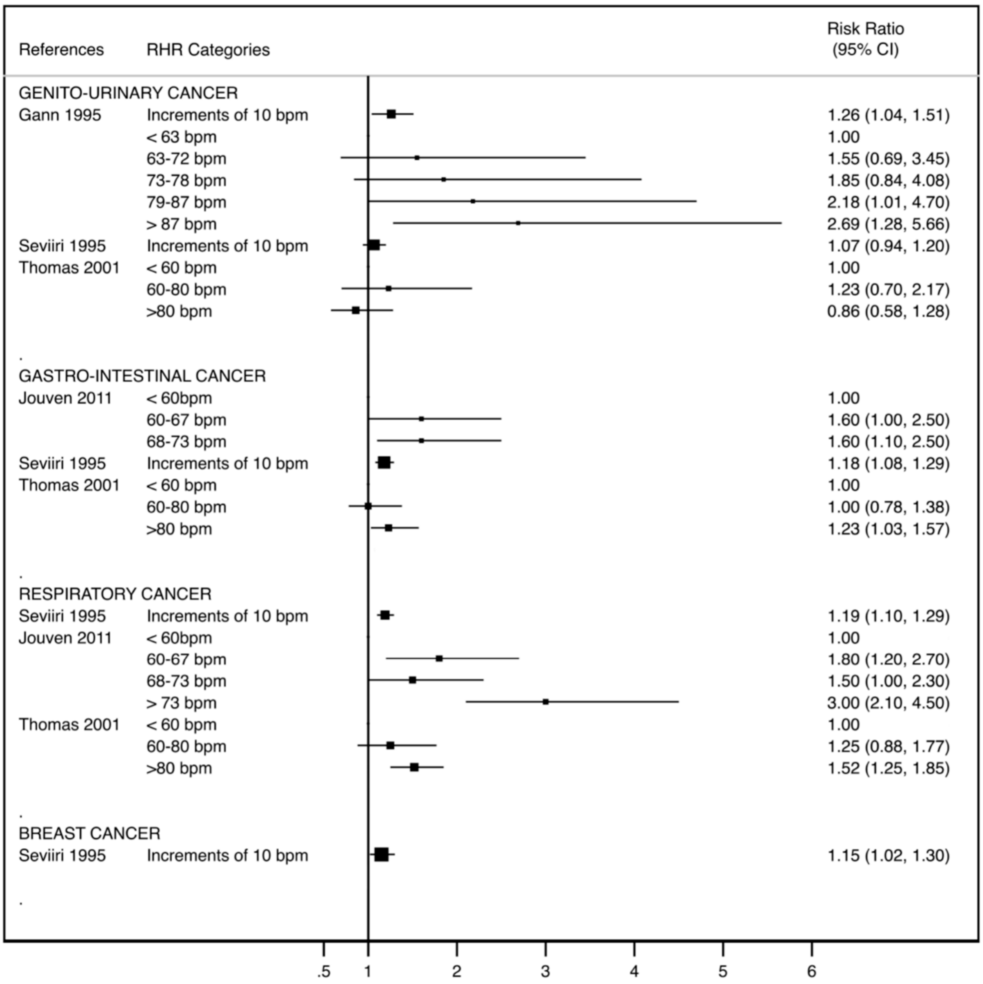

Supplement: Supplementary file 1 [file jcm-10-01354-s001.pdf]
